# Unsupervised Single-Channel Singing Voice Separation with Weighted Robust Principal Component Analysis Based on Gammatone Auditory Filterbank and Vocal Activity Detection

**DOI:** 10.3390/s23063015

**Published:** 2023-03-10

**Authors:** Feng Li, Yujun Hu, Lingling Wang

**Affiliations:** 1 Department of Computer Science and Technology, Anhui University of Finance and Economics, Bengbu 233030, China; 2 School of Information Science and Technology, University of Science and Technology of China, Hefei 230026, China

**Keywords:** single channel, singing voice, source separation, robust principal component analysis, gammatone filterbank, vocal activity detection

## Abstract

Singing-voice separation is a separation task that involves a singing voice and musical accompaniment. In this paper, we propose a novel, unsupervised methodology for extracting a singing voice from the background in a musical mixture. This method is a modification of robust principal component analysis (RPCA) that separates a singing voice by using weighting based on gammatone filterbank and vocal activity detection. Although RPCA is a helpful method for separating voices from the music mixture, it fails when one single value, such as drums, is much larger than others (e.g., the accompanying instruments). As a result, the proposed approach takes advantage of varying values between low-rank (background) and sparse matrices (singing voice). Additionally, we propose an expanded RPCA on the cochleagram by utilizing coalescent masking on the gammatone. Finally, we utilize vocal activity detection to enhance the separation outcomes by eliminating the lingering music signal. Evaluation results reveal that the proposed approach provides superior separation outcomes than RPCA on ccMixter and DSD100 datasets.

## 1. Introduction

Singing voice separation (SVS) has drawn a lot of interest and consideration in many downstream applications [1,2,3,4]. It deals with the technique of separating a singing voice or background from a mix of music, which is a crucial strategy for singer identification [5,6], music information retrieval [7,8], lyric recognition and alignment [9,10,11,12], song language identification [13,14], and chord recognition [15,16,17]. The recent separation techniques, however, fall well short of the capabilities of human hearing. It is challenging to resolve the existing SVS because of the instruments utilized and the spectral overlap between the speech and background music [11,18,19,20,21]. In daily life, human listeners generally have the remarkable ability to distinguish sound streams from a mixture of sounds, but this continues to be a difficult task for machines, particularly in the monaural case because it lacks the spatial cues that can be learned when two or more microphones are used. Additionally, the singing separation feeling could not directly translate from spoken separation. Speaking and singing voices have many similarities with one another but also differ in important ways. Because singing and speaking have distinct histories, there are significant challenges involved in separating them. The nature of the other accompanying sounds is the key distinction between singing and speech in terms of their independence from a background. The background noise that mingles with speech may be harmonic or nonharmonic, narrowband or broadband, and often unrelated to the speech. However, the musical accompaniment to a song is typically harmonic and wideband, associated with the singing, and does not adhere to the common misconceptions about noise, such as its whiteness or stationarity. Consequently, conventional noise-suppression techniques are inappropriate.

Furthermore, singing voices typically have distinct and powerful harmonic structures as well as harmonics that change quickly, such as vibratos or slides, and musical accompaniment may be thought of as the combination of percussive and harmonic elements. Because the extraction findings are erroneous because harmonic instruments (other than singing) also include harmonics, simple harmonic extraction techniques are still not suitable for polyphonic mixes and rapidly changing harmonics. Because singing voices and musical accompaniments typically start and stop at the same time, onset and offset cues—which are ordinarily helpful in auditory scene classification because distinct sounds typically start and end at various times—are also ineffective. Moreover, lyrics are conveyed through singing by altering notes in accordance with the melody, making singing an interpretation of a predetermined musical score. As a result, singing has a piecewise constant pitch with rapid pitch shifts and other sorts of variations. Until recently, various research strategies and algorithms have been introduced to improve the separation results in SVS tasks [22,23]. The deep learning techniques [24,25,26,27] are perhaps the most widely used for SVS. Yu et al. [24] proposed a new feature-extraction module based on UNet++ for SVS. An enhanced encoder–decoder is first created to initially extract multiscale information from a magnitude spectrogram of the mixed music. As a last step, soft masks are constructed for the separation of each source after more fine characteristics are obtained by using the feature-extraction module at various sizes. By utilizing the parameters it has trained, the suggested network can capture the key characteristics of the multiscale spectrogram. Hu et al. [25] proposed a hierarchic temporal convolutional network with cross-domain encoder for SVS. The model uses the hierarchic temporal convolutional network for the separation of different musical sources and integrates the complexed spectrogram domain feature and time-domain feature via a cross-domain encoder. With the help of the cross-domain encoder, the network will be able to encode interactive information for time-domain and complexed spectrogram domain characteristics. Guizzo et al. [26] proposed an antitransfer learning with convolutional neural network (CNN) for speech processing. Ni et al. [27] proposed a novel deep neural network based on UNet for SVS. The time-invariant, completely linked layers are built along the frequency axis of the network, which is a two-level nested U-structure. Due to its form, it is possible to record long-distance speech signal associations along the frequency alignment in addition to local and global context information. Also presented is a unique loss function that combines binary and ratio masks. The estimated voices channel is cleaner and carries fewer accompanying signals thanks to this technique.

Although they have been successful for SVS, these models present challenges when dealing with minimal audio data because a significant amount of training data is required beforehand [28,29,30,31,32]. Learning entails a lot of observation of the world’s objects and judgments made about their classifications, along with sporadic encounters with guided learning. In other words, knowledge gained from data may expand that learned from labeled data and may lead to the development of certain underlying assumptions or rules. Additionally, the limitations of previous neural networks become evident between the mismatch of training and testing samples [33]. The separation results lead to a decrease due to overfitting. In light of this, the unsupervised method is nevertheless appealing for mono source separation, especially when there is a lack of data or no other information. One of them is to assume and use the fundamental characteristics of singing voice and musical accompaniment. The RPCA strategy for SVS [34], which separates a mixture music into a singing voice (sparse) and background music (low rank), respectively. RPCA is a method that does not need any training or labeled data, and hence, it is convenient to use. It is the basis for or an inspiration for a number of unsupervised algorithms. Yang [35] developed two novel improvements of the matrix decomposition of the magnitude spectrogram by fusing the harmonicity priors information. Later, by breaking down a mixted spectrogram into a multiple low-rank representation (MLRR) will be introduced [36]. Despite being effectively applied to SVS, RPCA fails when one singular value, such as drums, is significantly greater than others, which lowers the separation results, particularly for drums included in the combined music signal. Although all approaches can produce effective separation results, they all ignore the characteristics of auditory system, which is crucial for enhancing the quality of separation outcomes. To overcome this problem, in previous studies, we proposed a novel unsupervised approached that extends RPCA exploiting rank-1 constraint for SVS tasking [37]. A recent study found that the cochleagram, a different time frequency (T-F) masking technique with gammatone, is more effective for audio signal separation than the spectrogram [38,39,40]. In the cochleagram representation, a gammatone filter is used to simulate the cochleagram representation’s frequency components, which are based on the human cochlea’s frequency selectivity ability. Yuan et al. [38] proposed a data augmentation method by using chromagram-based and pitch-aware methods for SVS. A popular and effective method for synchronizing and aligning music is the use of chromagrams or chroma-based characteristics. The twelve distinct pitch classes and chromagram are closely connected. In order to create a 1-D vector that represents how the harmonic content of the representation inside the timeframe is distributed throughout the 12 chroma bands, the fundamental concept is to aggregate each pitch class across octaves for a specific local time window. A 2-D time chroma representation is produced as the time frame is moved across the song. As a result of its great resilience to timbral fluctuations and tight relationship to the musical harmony, we employ a chromagram correlation across song sections as a metric by which to evaluate song commonalities. Gao et al. [39] proposed a novel machine learning method, the optimized nonnegative matrix factorization (NMF) for SVS. The suggested cost function was created specifically for factorization of nonstationary signals with temporally dependent frequency patterns. Moreover, He et al. [40] suggested a method that will be able to get around all of the sparse nonnegative matrix factorization (SNMF) 2-D’s previously mentioned drawbacks. The suggested model allows for many spectral and temporal changes, which are not inherent in the NMF and SNMF models. This allows for overcomplete representation. In order to provide distinctive and accurate representations of the nonstationary audio signals, sparsity must be imposed.

**Figure 1:**
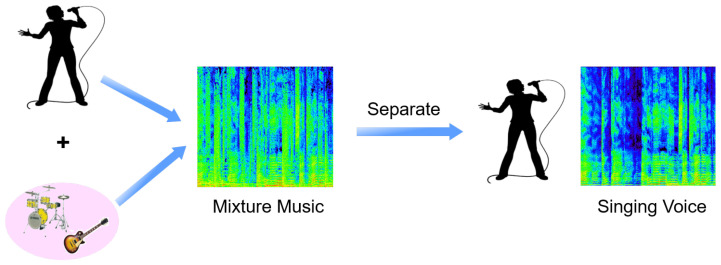
Blind monaural SVS system.

Additonally, the singing voice performances element on the cochleagram is rather distinct from the background music. For a singing voice, the spectral energy concentrates in a small number of time frequency units, so we may thus presume that it is sparse [41]. Additionally, the cochleagram’s accompaniment to music exhibits comparable patterns and structures that can be represented in the basis spectral vectors. As a result, an example of blind monaural SVS system is described in Figure 1. The underlying low-rank and sparsity hypotheses, however, could not always hold true. Both the decomposed low-rank matrix and the decomposed sparse matrix may include vocal sounds in addition to instrumental sounds (such as percussion). There is still some background music audible while listening to the separated singing voice. Similar to this, a portion of the singing voice is mistakenly categorized as background music. In order to improve the separation accuracy, additional approaches or techniques must be used to categorize the RPCA output.

Therefore, in this paper, to address the existing problems in RPCA for SVS, in our work, we provide the varying values approach to characterize low-rank and sparse matrices. This method is referred as weighted RPCA (WRPCA) [41], and it selects various weighted values from the separated by low-rank and sparse matrices. Meanwhile, as the first step of WRPCA, we simulate the human auditory system by using the gammatone filterbank. To further remove the nonseparated background music, we combined the harmonic masking and T-F masking [42,43,44]. The mixed signal’s time-frequency (T-F, or spectrogram) representation has been employed in the majority of prior speech-separation techniques. This signal is approximated from the waveform by using the short-time Fourier transform (STFT). The goal of speech separation technologies in the T-F domain is to approach the mixed spectrogram’s clean spectrogram of the separate sources. Nonlinear regression techniques may be used to directly approximate each source’s spectrogram description from the combination in this procedure. Finally, we utilize the vocal activity detection (VAD) [45,46,47] to get rid of any remaining background music. In a word, the key contributions of the work are outlined below as a summary.

- We offer the WRPCA addition to RPCA, which uses various weighted RPCA to achieve the improved separation performance.
- We combine gammatone auditory filterbank with vocal activity detection for SVS. Gammatone filterbanks are designed to imitate the human auditory system.
- We build the coalescent masking by fusing the harmonic masking and T-F masking, which can remove nonseparated background music. Additionally, we restrict the temporal segments that can include the singing voice part by using VAD.
- The extensive monaural SVS experiments reveal that the proposed approach can achieve greater separation performance than the RPCA method.

The remainder of this paper is arranged as follows. Section 2 provides RPCA and RPCA for SVS tasks. The proposed WRPCA on the cochleagram with VAD is illustrated in Section 3. The proposed approach is assessed with two different datasets in Section 4. Finally, we draw some conclusions from the paper and ideas for the further study in Section 5.

## 2. Related Work

This section discusses the RPCA and its application in SVS.

### 2.1. Overview of RPCA

RPCA was first introduced by Candés et al. [48] to divide the $M\in R_{m\times n}$ into $L\in R_{m\times n}$ plus $S\in R_{m\times n}$. Thus, the optimization model is defined as

(1) $$\begin{array}{c} \begin{array}{ccc} & minimize\;{\left|L\right|}_{*}+\lambda {\left|S\right|}_{1}, & \\ & subject\;to\;M=L+S. & , \end{array} \end{array}$$

where ${\left|L\right|}_{*}$ stands for the sum of singular values and ${\left|S\right|}_{1}$ is the sum of absolute values of the matrix. According to the previous study, we set $\lambda =1/\sqrt{max(m,n)}$. Meanwhile, we solve the convex program by accelerated proximal gradient (APG) or augmented Lagrange multiplier (ALM) [49] algorithms. In our work, a baseline experiment was conducted by using an inexact version of ALM.

### 2.2. RPCA for SVS

Music is typically made up of a variety of blended sounds, including both human singing voice and background music. The conventional RPCA approach can solve the task of SVS [34]. The magnitude spectrogram of a song may be broken down by using RPCA and can be thought of as the superposition of a sparse matrix and a low-rank matrix. The low-rank decomposition matrix and sparse matrix seem to match to the singing voice and background music. In light of these assumptions, RPCA may be used to solve the singing/accompaniment separation problem. The assumptions are that singing corresponds to sparse matrices and low rank to accompaniment.

**Figure 2:**
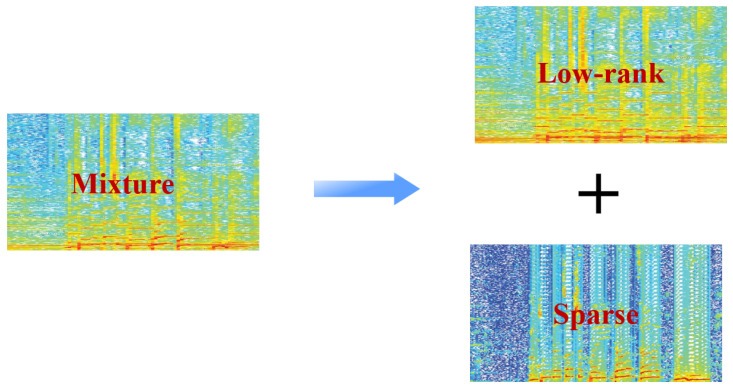
The separation process of SVS with low-rank and sparse model.

Due to the fact that musical instruments may replicate the same sounds again in music, the low-rank structure is used to conceptualize the spectrogram. In summary, the harmonic structure element of the singing voice part causes it to vary widely and to have a sparse distribution, producing in a spectrogram with the sparse matrix structure. Figure 2 shows the separation process of SVS with the low-rank and sparse model. Music is a low-rank signal because musical instruments can recreate the same sounds each time a piece is performed and music generally has an underlying recurring melodic pattern. Contrarily, voices are relatively scarce in the temporal and frequency domains but have greater diversity (higher rank). The singing voices can thus be seen as elements of the sparse matrix. By RPCA, we anticipate that the sparse matrix S will contain voice signals and the low-rank matrix L will include backing music.

Consequently, in this study, we may divide an input matrix by using the RPCA approach into a low-rank and sparse matrices. Nevertheless, it does make significant assumptions. Drums, for instance, could not be low rank but rather lie in the sparse subspace, which lowers the results, especially for drums included in mixed music.

## 3. Proposed Method

This section firstly presents the WRPCA approach. Then, the gammatone filterbank and vocal acivity detection are utilized as the postprocessing for SVS. Finally, we provide the architecture of the proposed SVS approach.

### 3.1. Overview of WRPCA

WRPCA is an extension of RPCA, which has different scale values between sparse and low-rank matrices. The corresponding model can be defined as follows,

(2) $$\begin{array}{c} \begin{array}{c} minimize\;\;{\left|L\right|}_{w,*}+\lambda {\left|S\right|}_{1},\\ subject\;to\;M=L+S. \end{array} \end{array}$$

where ${\left|L\right|}_{w,*}$ is the different weighted values in the matrix of low rank, and the *S* is sparse. $M\in R_{m\times n}$ is made up $L\in R_{m\times n}$ and $S\in R_{m\times n}$, and the parameter $\lambda =1/\sqrt{max(m,n)}$ is indicated [48]. Thus, we define the function of ${\left|L\right|}_{w,*}$ as follows,

(3) $$\begin{array}{c} {\left|L\right|}_{w,*}=\left|w_{i}\sigma_{i}\left(M\right)\right|, \end{array}$$

where $w_{i}$ denotes the weight assigned to singular value $\sigma_{i}\left(M\right)$.

In this paper, we also adopted an efficient, inexact version of the augmented Lagrange multiplier (ALM) [49] to solve this convex model. The corresponding augmented Lagrange function is defined as follows:

(4) $$\begin{array}{c} J\left(M,L,S,\mu \right)=\;\;{\left|L\right|}_{w,*}+\lambda \;{\left|S\right|}_{1}+<J,M-L-S>\\ +\frac{\mu }{2}{\left|M-L-S\right|}_{F}^{2}.\;\;\;\;\;\;\;\;\;\;\;\;\;\;\;\;\;\;\;\;\;\; \end{array}$$

where *J* is the Lagrange multiplier and µ is a positive scaler. The process corresponding to music mixture signal separation can be seen in Algorithm 1 WRPCA for SVS. The value of M is a mixture music signal from the observed data. After the separation by using WRPCA, we can obtain a sparse matrix S (singing voice) and a low-rank matrix L (music accompaniment).
**Algorithm 1** WRPCA for SVS**Input**: Mixture music $M\in R_{m\times n}$, weight w.1: **Initialize**: $\rho ,\mu_{0},L_{0}=M,J_{0}=0,k=0.$2: While not convergence **do**:3: **repeat**4:   $S_{k+1}=\;$arg$\min_{S}\;$${\left|S\right|}_{1}+\frac{\mu_{k}}{2}{\left|M+\mu_{k}^{-1}J_{k}-L_{k}-S\right|}_{F}^{2}.$5:   $L_{k+1}=\;$arg$\min_{L}\;$${\left|L\right|}_{w,*}+\frac{\mu_{k}}{2}{\left|M+\mu_{k}^{-1}J_{k}-S_{k+1}-L\right|}_{F}^{2}.$6:   $J_{k+1}=J_{k}+\mu_{k}\left(M-L_{k+1}-S_{k+1}\right).$7:   $\mu_{k+1}=\rho *\mu_{k}.$8: k←k+1.9: **end while.****Output**: $S_{m\times n},\;L_{m\times n}.$

### 3.2. Weighted Values

The standard nuclear norm minimization regularizes each singular value equally to pursue the convexity of the objective function. However, the RPCA method simply ignores the differences between the scales of the sparse and low-rank matrices. In order to solve this problem, and inspired by the success of weighted nuclear norm minimization [50], we adopted different weighted value strategies to trim the low-rank matrix during the SVS processing. This enables the features of the separated matrices to be better represented.

**Lemma** **1.** *Set M = $U\sum V^{T}$ as the singular value decomposition (SVD) of $M\in R_{m\times n}$, where*

(5) $$\begin{array}{c} \sum =\left(\begin{array}{c} diag(\delta_{1}\left(M\right),\delta_{2}\left(M\right),\ldots ,\delta_{n}\left(M\right))\\ \;\;\;\;\;\;\;\;\;\;\;\;\;\;\;\;\;\;0 \end{array}\right), \end{array}$$

*and $\delta_{i}\left(M\right)$ represents the i-th singular value of M.*

Thus, we define the weight function as follows,

(6) $$\begin{array}{c} W_{i}^{l+1}=\frac{C}{\delta_{i}\left(L_{l}\right)+\varepsilon }, \end{array}$$

where ε is a small positive number to avoid dividing by zero and *C* is a compromising constant. *C* > 0 and 0 < $\varepsilon <min(\sqrt{C},\frac{C}{\delta_{1}\left(M\right)})$. According to the enhancing sparsity by reweighed $l_{1}$ minimization [51], the reweighted model is described as follows,

(7) $$\begin{array}{c} L^{*}=U\sum^{\prime }V^{T}, \end{array}$$

where

(8) $$\begin{array}{c} \sum^{\prime }=\left(\begin{array}{c} diag(\delta_{1}\left(L^{*}\right),\delta_{2}\left(L^{*}\right),\ldots ,\delta_{n}\left(L^{*}\right))\\ \;\;\;\;\;\;\;\;\;\;\;\;\;\;\;\;\;\;0 \end{array}\right), \end{array}$$

and

(9) $$\begin{array}{c} \delta_{i}\left(L^{*}\right)=\left\{\begin{array}{cccc} & \;\;\;\;0 & \;\; & \;\;\\ & \frac{c_{1}+\sqrt{c_{2}}}{2}, & \;\; & \;\; \end{array}\right. \end{array}$$

where $c_{1}=\delta_{i}\left(M\right)-\varepsilon$ and $c_{2}={\left(\delta_{i}\left(M\right)+\varepsilon \right)}^{2}-4C$. In this paper, the maximum matrix size was determined empirically by the regularization parameter C=max(m,n) [50].

### 3.3. Gammatone Filterbank

The information obtained from primary auditory fibers is characterized by the gammatone function [52]. It characterizes physiological impulse-response data gathered from primary auditory fibers in the cat. Gammatone filter banks were designed to model the human auditory system. The modeling process mimics the organization of the peripheral sound processing step cites using a physiologically strategy [53]. In our work, we first pass a mixture music signal into the gammatone filterbank. Thus, the impulse response function is defined as follows,

(10) $$\begin{array}{c} h\left(t\right)=At^{N-1}\exp\left(-2\pi bt\right)\cos\left(2\pi f_{c}t+\varphi \right)\;\left(t\geq 0,N\geq 1\right), \end{array}$$

where *A* is an arbitrary factor, *N* is the filter order, *b* is the between the impulse functions’length and the filter’s bandwidth, $f_{c}$ is the center frequency, and φ is the tone phase.

**Figure 3:**
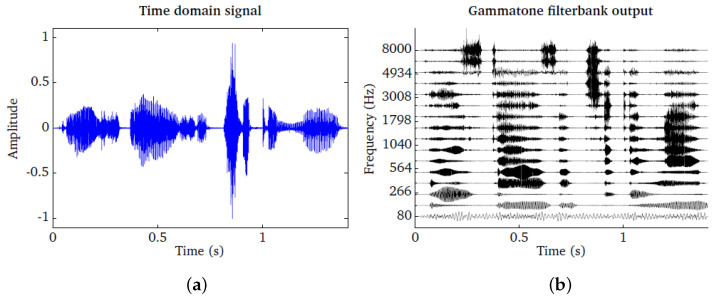
(**a**) Time domain signal. (**b**) The corresponding output of gammatone.

In the human auditory system, there are around 3000 inner hair cells along the 35-mm spiral path cochlea. Each hair cell could resonate to a certain frequency within a suitable critical bandwidth. This means that there are approximately 3000 bandpass filters in the human auditory system. This high resolution of filters can be approximated by specifying certain overlapping between the contiguous filters. The impulse response of each filter follows the gammatone function shape. The bandwidth of each filter is determined according to the auditory critical band, which is the bandwidth of the human auditory filter at different characteristic frequencies along the cochlea path [54]. The speech signal shown in the left panel is passed through a bank of 16 gammatone filters spaced between 80 Hz and 8000 Hz. The output of each individual filter is shown in the right panel. As a result, Figure 3 depicts the gammatone filterbank.

### 3.4. T-F Masking

After obtaining the separation results of sparse *S* and low-rank matrices *L* by using WRPCA, we applied T-F masking to further improve the separation performance. Thus, we define the ideal binary mask (IBM) and ideal ratio mask (IRM) as follows,

(11) $$\begin{array}{c} IBM=\left\{\begin{array}{cccc} & 1 & \;\;\;S_{i,j}\geq L_{i,j} & \;\;\\ & 0 & \;\;\;S_{i,j}<L_{i,j}, & \;\; \end{array}\right. \end{array}$$

and

(12) $$\begin{array}{c} IRM=\frac{S_{i,j}}{S_{i,j}+L_{i,j}} \end{array}$$

where $S_{i,j}$ and $L_{i,j}$ denote the complex spectral values of singing voice and accompaniment, respectively.

### 3.5. F0 Estimation

In this work, we use F0 to enhance the effectiveness of separation results. Due to the fact that F0 varies over time and is a property of the parts played by various singing voice and background accompaniment, it can greatly improve separation quality by removing the spectral components of nonrepeating instruments (e.g., bass and guitar). The salience function is defined as

(13) $$\begin{array}{c} H\left(t,s\right)=\sum_{n=1}^{N}h_{n}P\left(t,s+1200\log_{2}\left(n\right)\right), \end{array}$$

where *t* is the sequence index and *s* is the logarithmic frequency. The number of harmonic components is *N* and the decaying fact is $h_{n}$.

The function of *C* can be calculated as

(14) $$\begin{array}{c} C=argmax\sum_{t=1}^{T-1}\left(\log a_{t}H\left(t,s_{t}\right)+\log T\left(s_{t},s_{t+1}\right)\right), \end{array}$$

where $a_{t}$ is the factor in normalization that brings the salience values to a sum of 1, and $T(s_{t},s_{t+1})$ is a transition probability that denotes the likelihood of current F0 moving to the next F0 in the following sequence. Additionally, by utilizing the Viterbi search approach, the melody contour *C* value is optimized.

### 3.6. Harmonic Masking

As a result of our prior study [55], the harmonic masking is defined as

(15) $$\begin{array}{c} M_{h}\left(t,f\right)=\left\{\begin{array}{cccc} & \;\;1 & \;\;\;nF_{t}-\frac{w}{2}<f<nF_{t}+\frac{w}{2} & \;\;\\ & \;\;0 & \;\;\;others, & \;\; \end{array}\right. \end{array}$$

where *w* is the frequency width used to extract the energy surrounding each harmonic, *n* is the harmonic’s index, and the vocal F0 is represented by $F_{t}$ at sequence *t*.

### 3.7. Coalescent Masking

We are interested in constructing coalescent masking by using harmonic masking $M_{h}$ and IBM. It is possible to define the corresponding formulation $M_{c}$ as follows,

(16) $$\begin{array}{c} M_{c}=IBM⊗M_{h}, \end{array}$$

where the elementwise multiplication operator is indicated by ⊗, and the time frequency masking and harmonic masking are denoted by IBM and $M_{h}$, respectively.

### 3.8. Vocal Activity Detection

To remove the residual music signal and restrict the values of voice and accompaniment, we are using a VAD approach. The output results $s_{o}$ can be described as follows,

(17) $$\begin{array}{c} s_{o}=\left\{\begin{array}{cccc} & s_{v} & \;\;\;\Omega >k & \;\;\\ & s_{a} & \;\;\;others, & \;\; \end{array}\right. \end{array}$$

where $s_{v}$ is the state of the singing voice, $s_{a}$ is the state of the background music, and *k* is the threshold. According to the vocal F0 estimation methods [56], the definition of the function Ω is as follows,

(18) $$\begin{array}{c} \Omega =\sum_{f}{\left\{\frac{1}{H_{f}}\sum_{n=1}^{H_{f}}P\left(t,s+1200\log_{2}^{n}\right)\right\}}^{1.8}, \end{array}$$

where P(t,s) denotes the the value of power and $H_{f}$ is the sum of harmonics for each frequency.

**Figure 4:**
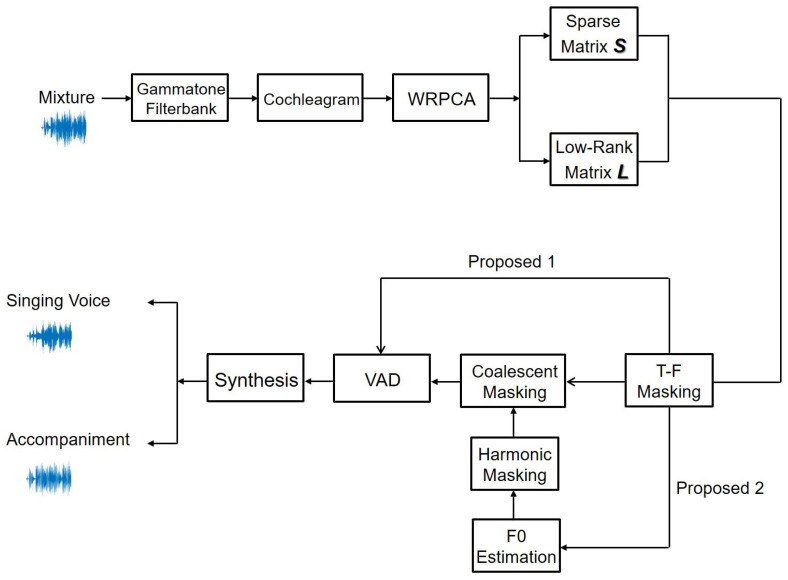
The architecture of the proposed SVS approach.

The architecture of our proposed method for the blind monaural SVS system is illustrated in Figure 4. We first apply a gammatone filterbank to the test dataset’s mixture music signal to obtain the cochleagram, and then utilize proposed WRPCA approach to separate it into the *L* and *S*. By combining T-F masking and harmonic masking, we also create coalescent masking to eliminate nonseparated music. To enhance separation performance, VAD is being used. Finally, we synthesize the voice and background music. According to Wang et al. [57], the separated signal can be synthesized.

**Figure 5:**
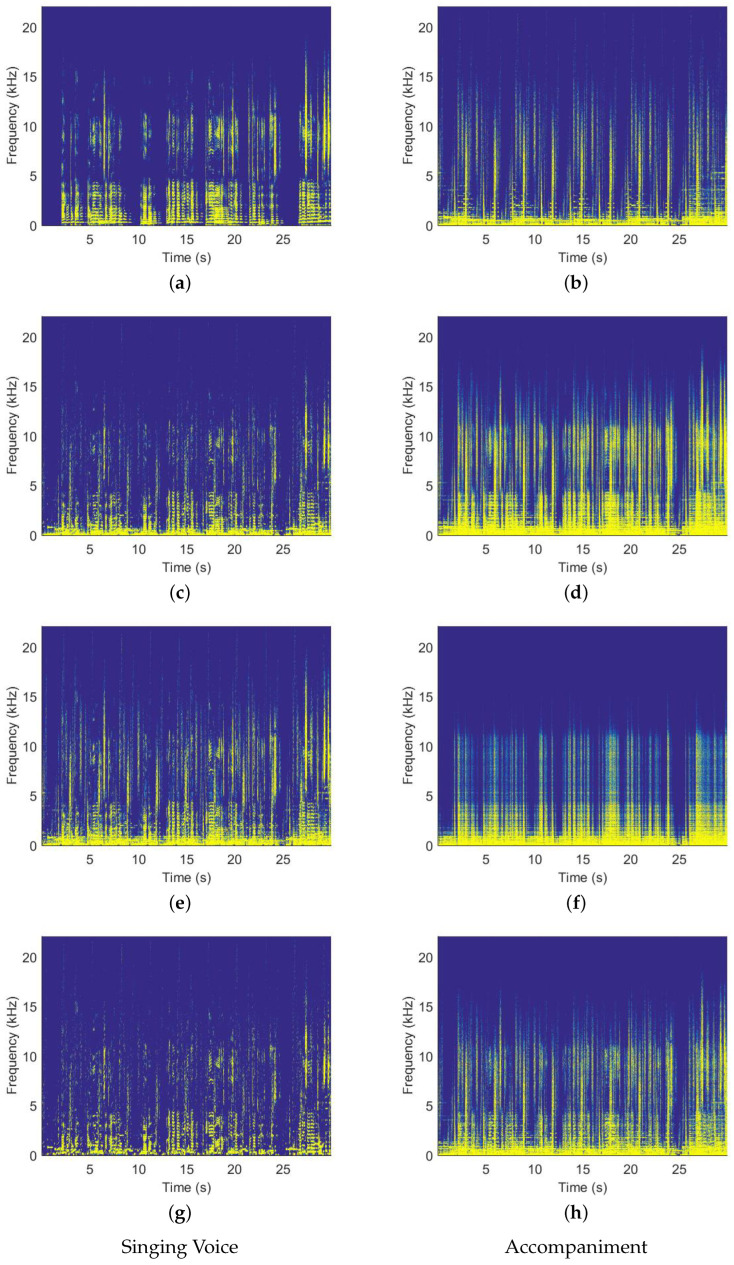
Examples are taken from the ccMixter dataset’s spectrograms. The singing voice is represented by the left four spectrograms, whereas the equivalent musical accompaniment is represented by the right four.

In this work, we randomly selected 30-s audio sample data at random in the ccMixter. The spectrograms of the isolated singing voice and background portions from mixed musical signals are illustrated in Figure 5. The original spectrograms are contrasted employing different separation approaches. As seen in the figures, the original clean singing voice and music spectrograms are shown in (a) and (b), whereas (c) and (d) exhibit the separated signal divided by RPCA. The WRPCA has split the signals (e) and (f) in the **Proposed 1**. Similarly, (g) and (h) show the separation results by the **Proposed 2**.

From the abovementioned spectrograms, we can see that Figure 5c has the strongest background music signal (accompaniment), whereas Figure 5g has the lowest constraint. In other words, the latter is therefore preferable than the former in processing of SVS.

## 4. Experimental Evaluation

This section discusses the two experiments for SVS, including datasets we used on ccMixter [58] and DSD100 [59], respectively. We also present a comparison and analysis of the experiment results.

### 4.1. Datasets

One was the ccMixter, for which we selected 43 full stereo tracks with only 30 s (from 30 s to 1 min) at the same time of each track, and every piece of music can only contain voice for so long. Three components make up each mixture song: voice, background and the combination.

Another was the DSD100 dataset, which consisted of 36 development data and 46 test data. To reduce dimensionality and speed up speech processing, we also utilized 30-s fragments (from 1 min 45 s to 2 min 15 s) simultaneously for all data.

### 4.2. Settings

In our work, we focused on single-channel source separation, which is more difficult and complex than multichannel source separation because only less useful information is available. The two-channel stereo mixture datasets we used were downmixed to be mono by averaging two channels.

To evaluate our proposed approach, the spectrogram was computed by STFT by using 1024 points, and the size of hope is 256 samples. The experimental data was converted to mono after being sampled at 44.1 kHz. We established 128 channels, the frequency length ranged from 40 to 11,025 Hz, and a 256 frequency length for cochleagram analysis.

To confirm the effectiveness of our proposed algorithm, we assessed its quality of separation in terms of the source-to-distortion ratio (SDR) and the source-to-artifact ratio (SAR) by using the BSS-EVAL 3.0 metrics [60,61] and the normalized SDR (NSDR). Therefore, we define the estimated value $\hat{S}$(*t*) as follows,

(19) $$\hat{S}\left(t\right)=S_{target}\left(t\right)+S_{interf}\left(t\right)+S_{artif}\left(t\right),$$

where $S_{target}\left(t\right)$ is the target audio’s permissible distortion, $S_{interf}\left(t\right)$ denotes the allowable length change of source information to account for disturbances from unwanted sources, and $S_{artif}\left(t\right)$ denotes a potential artifact connected to the artifact of the separation technique. We therefore categorize them as follows,

(20) $$SDR=10log_{10}\frac{\sum_{t}S_{target}{\left(t\right)}^{2}}{\sum_{t}{\left\{S_{interf}\left(t\right)+S_{artif}\left(t\right)\right\}}^{2}},\;\;$$

(21) $$SAR=10log_{10}\frac{\sum_{t}{\left\{S_{target}\left(t\right)+S_{interf}\left(t\right)\right\}}^{2}}{\sum_{t}S_{artif}{\left(t\right)}^{2}},$$

(22) $$NSDR\left(\hat{v},v,x\right)=SDR\left(\hat{v},v\right)-SDR\left(x,v\right),\;\;\;\;$$

where $\hat{v}$ is the estimated signal, *v* stands for the reference isolated, and *x* for the mixed music. The NSDR takes into account the SDR’s overall increase from *x* to $\hat{v}$. The measurement units used are all dB.

The higher values of the SDR, SAR and NSDR represent that the method exhibits better separation performance of source separation. The SDR represents the quality of the separated target sound signals. The SAR represents the absence of artificial distortion. All the metrics are expressed in dB.

### 4.3. Experiment Results

The following two approaches are presented based on the proposed WRPCA; we take them as Proposed 1 and Proposed 2, respectively. More specifically, the Proposed 1 is utilize WRPCA and T-F masking, whereas Proposed 2 adopts WRPCA and coalescent masking. Both algorithms are use VAD technology:

- **Proposed 1**: WRPCA with T-F masking
- **Proposed 2**: WRPCA with coalescent masking.

**Figure 6:**
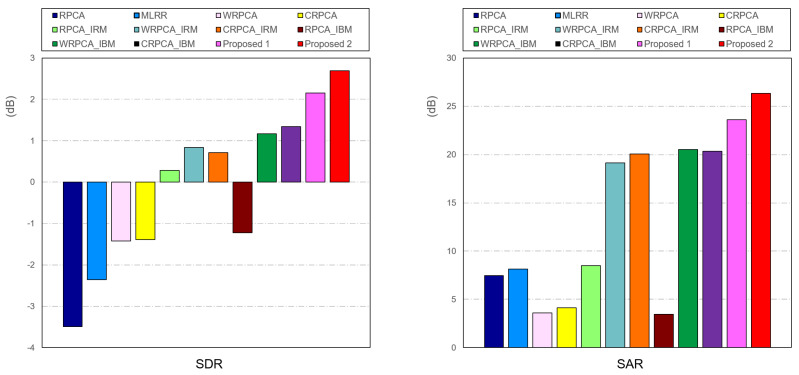
Comparison of SVS on **ccMixter** dataset for each of the following: RPCA, MLRR, WRPCA, CRPCA, RPCA using IRM, WRPCA using IRM, WRPCA using IRM, RPCA using IBM, WRPCA using IBM, CRPCA using IBM, Proposed 1, and Proposed 2, respectively.

We evaluated them by using the ccMixter. The comparative outcomes for RPCA, MLRR, WRPCA, CRPCA, RPCA with IRM, WRPCA using IRM, CRPCA using IRM, RPCA using IBM, WRPCA using IBM, CRPCA using IBM, Proposed 1, and Proposed 2 are shown in Figure 6. To further completely confirm the efficacy of our proposed methodology, we designed multiple comparative experiments. The RPCA, MLRR, WRPCA, CRPCA, RPCA using IRM, RPCA using IBM, CRPCA using IRM, and CRPCA using IBM are evaluated on the spectrogram, whereas the WRPCA using IRM, WRPCA using IBM, Proposed 1, and Proposed 2 are evaluated on the cochleagram. We can find from the SDR and SAR experiment results that WRPCA performs better, especially for VAD on the cochleagram. The standard RPCA, in contrast, performed less well than the others.

**Figure 7:**
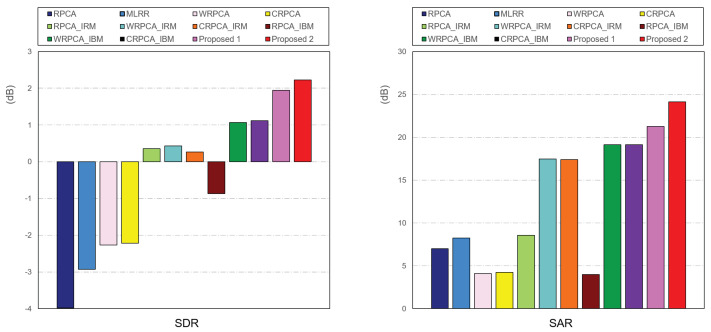
Comparison of SVS on **DSD100** dataset for each of the following: RPCA, MLRR, WRPCA, CRPCA, RPCA using IRM, WRPCA using IRM, WRPCA using IRM, RPCA using IBM, WRPCA using IBM, CRPCA using IBM, Proposed 1, and Proposed 2, respectively.

Additionally, we assessed WRPCA by using the DSD100 dataset. The comparative outcomes for RPCA, MLRR, WRPCA, CRPCA, RPCA using IRM, WRPCA using IRM, CRPCA using IRM, RPCA using IBM, WRPCA using IBM, CRPCA using IRM, Proposed 1, and Proposed 2 are each shown in Figure 7. Similarly, RPCA, WRPCA, CRPCA, RPCA using IRM, RPCA using IBM, CRPCA using IRM, and CRPCA using IBM are evaluated on the spectrogram, whereas the WRPCA using IRM, WRPCA using IBM, Proposed 1, and Proposed 2 are evaluated with the cochleagram. We can find from the SDR and SAR experiment results that WRPCA performs better, especially for the VAD on the cochleagram. The standard RPCA, in contrast, performed less well than the others in Figure 6 and Figure 7, respectively.

**Figure 8:**
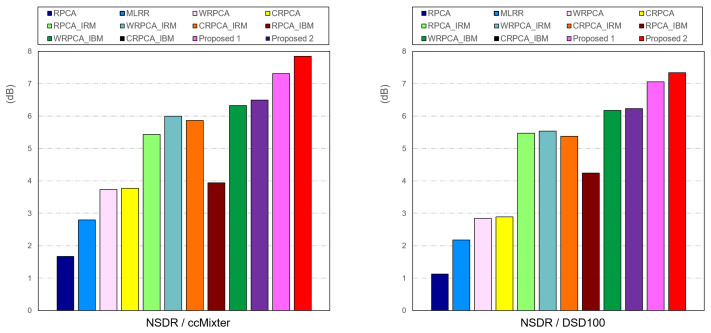
Comparison of SVS between **ccMixter** and **DSD100** datasets for each of the following: RPCA, MLRR, WRPCA, CRPCA, RPCA using IRM, WRPCA using IRM, WRPCA using IRM, RPCA using IBM, WRPCA using IBM, CRPCA using IBM, Proposed 1, and Proposed 2, respectively.

Figure 8 exhibits the NSDR results from the ccMixter and DSD100 datasets that we obtained by using WRPCA. In other words, the NSDR gives improved removal efficiency in SVS and overall optimizes the SDR. The results demonstrated that our Proposed 2, which was used, produced the best results.

As a consequence, we confirm that WRPCA on a cochleagram offers higher sensitivity and selectivity than RPCA under similar circumstances with or without T-F masking based on the results of Figure 6, Figure 7, and Figure 8, respectively. Additionally, WRPCA delivered superior outcomes to RPCA by using the gammatone and T-F masking. We show that, across all evaluation modalities, our suggested strategies offer improved separation outcomes.

## 5. Conclusions

In this work, we proposed an extension of RPCA by using weighting on the cochleagram. The mixing signal’s cochleagram was segmented into low-rank and sparse matrices by WRPCA, and the coalescent masking was constructed by integrating the harmonic and T-F masking. Finally, we constrained the temporal segments that could include the singing voice part utilizing VAD. Evaluations on ccMixter and DSD100 datasets reveal that WRPCA performs better than RPCA for SVS, especially for WRPCA on cochleagram using gammatone and VAD approach.

In future work, to further expand the functionality of our system, we will research the vocal augmentation option. Additionally, unsupervised training that depends on the complementary nature of these two tasks will be tried because of the modest size of the public datasets that comprise both pure vocal samples and their related F0 annotations.

## Data Availability

The datasets generated during and analysed during the current study are available from the corresponding author on reasonable request.

## Abbreviations

The following abbreviations are used in this paper:

SVS	singing voice separation
RPCA	robust principal component analysis
MLRR	multiple low-rank representation
WRPCA	weighted robust principal component analysis
VAD	voice activity detection
IBM	ideal binary mask
IRM	ideal ratio mask
ALM	augmented lagrange multiplier
APG	accelerated proximal gradient
SVD	singular value decomposition
T-F	time-frequency
CNN	convolutional neural network
NMF	nonnegative matrix factorization
SNMF	sparse nonnegative matrix factorization
SDR	source-to-distortion ratio
SAR	source-to-artifact ratio
NSDR	normalized SDR
